# Biochemical Characterization of a Novel Bacterial Laccase and Improvement of Its Efficiency by Directed Evolution on Dye Degradation

**DOI:** 10.3389/fmicb.2021.633004

**Published:** 2021-05-12

**Authors:** Shuang Dai, Qian Yao, Gen Yu, Shan Liu, Jeonyun Yun, Xiong Xiao, Zujun Deng, He Li

**Affiliations:** ^1^Guangdong Key Laboratory of Pharmaceutical Bioactive Substances, College of Life Science and Biopharmaceuticals, Guangdong Pharmaceutical University, Guangzhou, China; ^2^Guangzhou Base Clean Cosmetics Manufacturer Co., Ltd., Guangzhou, China

**Keywords:** laccase, dye degradation, directed evolution, enzymatic characteristics, expression

## Abstract

Laccase is a copper-containing polyphenol oxidase with a wide range of substrates, possessing a good application prospect in wastewater treatment and dye degradation. The purpose of this research is to study the degradation of various industrial dyes by recombinant laccase rlac1338 and the mutant enzyme lac2-9 with the highest enzyme activity after modification by error-prone PCR. Four enzyme activities improved mutant enzymes were obtained through preliminary screening and rescreening, of which lac2-9 has the highest enzyme activity. There are four mutation sites, including V281A, V281A, P309L, S318G, and D232V. The results showed that the expression of the optimized mutant enzyme also increased by 22 ± 2% compared to the unoptimized enzyme and the optimal reaction temperature of the mutant enzyme lac2-9 was 5°C higher than that of the rlac1338, and the optimal pH increased by 0.5 units. The thermal stability and pH stability of mutant enzyme lac2-9 were also improved. With ABTS as the substrate, the k_cat_/K_m_ of rlac1338 and mutant strain lac2-9 are the largest than other substrates, 0.1638 and 0.618 s^–1^M^–1^, respectively, indicating that ABTS is the most suitable substrate for the recombinant enzyme and mutant enzyme. In addition, the K_m_ of the mutant strain lac2-9 (76 μM) was significantly lower, but the k_cat_/K_m_ (0.618 s^–1^M^–1^) was significantly higher, and the specific enzyme activity (79.8 U/mg) increased by 3.5 times compared with the recombinant laccase (22.8 U/mg). The dye degradation results showed that the use of rlac1338 and lac2-9 alone had no degradation effect on the industrial dyes [indigo, amaranth, bromophenol blue, acid violet 7, Congo red, coomassie brilliant blue (G250)], however, adding small molecular mediators Ca^2+^ and ABTS at the same time can significantly improve the degradation ability. Compared to the rlac1338, the degradation rates with the simultaneous addition of Ca^2+^ and ABTS of mutant enzyme lac2-9 for acid violet 7, bromophenol blue and coomassie brilliant blue significantly improved by 8.3; 3.4 and 3.4 times. Therefore, the results indicated that the error-prone PCR was a feasible method to improve the degradation activity of laccase for environmental pollutants, which provided a basis for the application of laccase on dye degradation and other environmental pollutants.

## Introduction

Laccase, first discovered in the sap of the Japanese lacquer tree *Rhus vernicefera* (Wang and Chen, 2003) is one of the earliest enzymes studied. It belongs to the copper oxidase family. Laccase can use O_2_ to oxidize various aromatics and non-aromatic compounds through free radical catalytic reactions. Laccase has a wide range of sources, mainly from plants, fungi, and bacteria. With the gradual discovery of the function of laccase in dye degradation, the research on laccase has received extensive attention.

At present, there are more than 10,000 industrial dyes, according to the composition, it can be divided into azo, indigo, anthraquinone and triphenylmethane dyes. The mechanism of laccase on dyes from different sources is very different (Zhang et al., 2017). Laccase can directly oxidize anthraquinone dyes (Li, 2013) and azo dyes (Si et al., 2011); for some recalcitrant dyes, laccase often requires the participation of small molecular mediators to achieve efficient decolorization. For example, Susana et al. (2007) used the laccase mediator system to degrade reactive black and sky blue, the results showed that the laccase mediator system had a degradation rate of 90% for reactive black within 30 min, and the degradation rate for sky blue was also up to 30%, and the laccase system alone has almost no degradation effect on these two dyes.

At present, enzyme modification methods are mainly grouped into two categories, namely rational design methods and non-rational design methods (Mills et al., 1967). Among them, non-rational design does not need to understand the relationship between protein structure and function. By simulating the process of natural evolution in the laboratory, after random mutation, recombination and selection, the long natural evolution process can be simulated in a short time. The error-prone PCR (error-prone PCR) selected in this experiment is a non-rational design method, which can carry out directed evolution and selection of the coding genes of enzyme molecules, thereby improving the stability of the enzyme and the specificity of the substrate. It is an important research tool to the current protein engineering. The *in vitro* molecular directed evolution technology based on error-prone PCR can produce large phenotypic differences through minimal sequence changes, and target strains can be screened out, which simplifies the comparative analysis of sequences to a great extent. However, because error-prone PCR can only mutate a small sequence in the original protein, it is generally suitable for smaller gene fragments (<2,000 bp) (Wang et al., 2017).

In this study, a laccase gene *lac1338* from the marine microbial metagenome was synthesized by a codon optimization for *E. coli* expression. Then the laccase rlac1338 with high expression and thermal stability was obtained through prokaryotic expression. The laccase can degrade various dyes, but due to its low enzymatic activity than comparable bacterial or fungal laccases, the dye degradation rates are also low, which limits its application to a certain extent. Therefore, we modified the *lac1338* gene by an error-prone PCR directed evolution strategy, and used enzyme activity as a screening indicator to obtain mutant strains with increased laccase activity. By comparing the optimum temperature and temperature stability, optimum pH and pH stability, enzymatic kinetics, and dye degradation rate between the recombinase and the mutant enzyme, we have determined that error-prone PCR modification of laccase is a feasible method. Further explore its application prospects in industrial dye processing, and provide a certain foundation for its suitability for industrial applications.

## Materials and Methods

### Strains and Plasmids

The recombinant plasmid pUC118-lac1338 (GenBank, accession number HM623889) was constructed and stored in our laboratory. pET-32a (+) and *Escherichia coli* BL2l (DE3) were purchased from Novagen.

### Main Reagents

A gel recovery kit, a plasmid extraction kit, and a PCR product recovery kit (all from Omega), a protein purification kit (Novagen, Germany), and a Diversify^®^ PCR random mutagenesis kit (Clontech). A 170 kD prestained protein ladder was purchased from Guangzhou Saizhe Biotechnology Co., Ltd. Other reagents included ABTS (Amresco), IPTG and ampicillin (both TaKaRa). Other conventional reagents were all analytically pure.

### Recombinant Plasmid Construction and Protein Expression

According to the sequencing results, primer pairs were designed on Premier 5:

lac1338-F: 5′-CCGGAATTCATGCGCAAAAGTCCCGGAGT CACTTTTTCA-3′ (the underlined part is the *Bam*H I restriction site);
lac1338-R: 5′-AGCAAGCTTTCAGTCGGGCATGTTGGGGA TTTCAGG-3′ (the underlined part is the *Hind* III restriction site).

Diversify^®^ PCR random mutagenesis kit was used for PCR amplification. *Bam*H I and *Hind* III restriction sites were added at both ends of the sequence, ligated to the *Bam*H I and *Hind* III sites of the expression vector pET-32a(+), the recombinant plasmid was obtained and verified by double enzyme digestion. The recombinant plasmid pET-32a-lac1338 was transformed into *E. coli* BL21(DE3) and placed under shaking culture at 37°C and 200 rpm/min until the OD_600_ reached about 0.8. After that, (Isopropyl-β-D-thiogalactopyranoside), and 0.5 mmol/L Cu^2+^ (final concentrations) were added, followed by incubation at 30°C and 200 rpm/min under shaking for 16 h to induce protein expression, centrifuge the bacteria to collect the precipitate and ultrasonically break it. Referred to the instruction of His⋅Bind^®^ Purification Kit (Novagen) for protein purification and SDS-PAGE analysis. With bovine serum albumin (BSA) as the standard, the purified protein concentration was determined using the bicinchoninic acid (BCA) method (Brown et al., 1989).

### Directed Evolution of lac1338

With lac1338 as the template, error-prone PCR was performed according to the instruction of the Diversify^®^ PCR random mutagenesis kit. A mutation rate of 2.3 bp/kb was selected, and the reaction system was: 10 × TITANIUM Taq Buffer 5 μl; 50 × Diversify dNTP Mix 1 μl; Template DNA (∼1 ng/μl) 1 μl; Mut-F (250 ng/μl) 1 μl; Mut-R (250 ng/μl) 1 μl; TITANIUM Taq Polym 1 μl; MnSO4 1 μl; dGTP (2 mM) 1 μl; PCR Grade Water 39 μl. The reaction conditions were: 94°C 30 s; 94°C 30 s, 68°C 90 s, 25 cycles; 72°C 5 min. The error-prone PCR product was cloned and constructed into *E. coli* BL21 (DE3) and the mutants with the highest enzyme activity were selected for experimental research through preliminary screening and re-screening of highly active laccase mutants. Among them, the initial screening was through the ABTS color reaction, and the enzyme activity was judged according to the color depth; the second screening was to identify whether the size of the mutant laccase was consistent with the size of the recombinant laccase by SDS-PAGE gel electrophoresis, and then selected by the enzyme activity determination method the mutant enzyme with the highest enzyme activity (Feng and Li, 2015).

### Activity Determination and Enzymatic Properties of Recombinant Laccase rlac1338 and Mutant Strain lac2-9

#### Enzyme Activity Determination

With ABTS as the substrate, the reaction system of 3 mL citric acid-sodium citrate buffer solution contains 5 mmol/L ABTS, 6 mmol/L Cu^2+^ and a certain concentration of the enzyme solution. After uniform mixing, the sample system reacted in a water bath at 55°C for 3 min, followed by measurement of OD_405_, measured three times in parallel and repeated the experiment three times. A blank control was prepared without adding the enzyme solution. Under these conditions, the amount of enzyme required to catalyze the oxidation of 1 μmol ABTS per minute was defined as 1 enzyme activity unit (U).

#### Determination of Optimal Reaction Conditions and Stability Analysis

The optimal reaction temperature was determined by measuring the enzyme activity from 30 to 70°C (5°C interval). The optimal reaction pH of the enzyme was identified within pH 4–10 (Britton-Robinson buffer solution). To determine the thermostability of the enzyme, its activity under the optimum pH was measured from 35 to 80°C (5°C interval) for 2 h. The enzyme activity of the enzyme solution stored at 4°C was considered as 100%. To evaluate the pH stability of the enzyme, the activity at the optimum temperature was measured after 4 h of incubation at pH of 4–8. The activity of the untreated enzyme solution was taken as 100%.

#### Determination of Kinetic Parameters of Enzymatic Reaction of Recombinant Laccase rlac1338 and Mutant Strain lac2-9

The Michaelis constant K_m_ and the maximum rate V_max_ were determined:

Different common substrates of laccase were selected (2,6-DMP, ABTS, Guaiacol, Catecho, 1-naphthol) to react. The OD_405_ was determined to calculate the initial speed of the enzyme reaction and plot a Lineweaver-Burk double reciprocal graph.

### Comparison of Dye Degradation by rlac1338 and lac2-9

The effects of rlac1338 and mutant enzyme lac2-9 on the degradation of various industrial dyes (amaranth, isatin, bromophenol blue, acid violet 7, crystal violet, orange red G, Congo red, rhodamine B, methylene blue and coomassie brilliant blue G250) were investigated. Inoculated 0.2 mL of recombinant laccase rlac1338 and mutant enzyme lac2-9, respectively, in 50 mL of liquid medium, and kept them at the optimum temperature (55, 60°C) and the suitable pH (6, 6.5), shook overnight at 220 rpm. After 16 h, added the above industrial dyes to make the final concentration of the dye 100 mg/L. After decolorizing at 37°C and 220 rpm for 24 h, the absorbance of each dye solution was determined at the maximum wavelength of the spectrophotometer. Then the effects of Ca^2+^ and ABTS on the degradation rate were simultaneously investigated.

Degradation⁢rate⁢(I)⁢was⁢calculated⁢as⁢I=

$$\left(A_{0}-A_{1}\right)/A_{0}\times 100\%,$$

where A_0_: light absorption of the blank control; A_1_: light absorption of a sample dye.

### Simulation of the Three-Dimensional Structure of the Mutant Strain

The amino acid sequence of the target protein was inputted into PHYRE2^1^ to predict structure. The PDB file was opened with Pymol, and the mutation site was marked. According to the predicted structure information, the site mutation effect was analyzed together with a comparison of enzymatic properties between the mutant enzyme and the wild-type enzyme.

## Results

### Recombinant Plasmid Construction and Protein Expression

The 1,338 bp *lac1338* gene was amplified by PCR (Supplementary Figure 1) and double enzyme digestion resulted in two bands of ∼1,338 and 5,900 bp (Supplementary Figure 2), respectively, which were the target gene and the empty linear plasmid pET-32a(+), which proved that the target gene was successfully ligated to the expression vector. SDS-PAGE showed that the rlac1338 was a single band with a molecular weight of ∼68 kD (18 kD was the fusion protein tag on the expression vector) (Supplementary Figure 3), which was the same as the theoretically predicted protein molecular weight. The purified protein concentration measured by the BCA method was 0.25 g/L.

### Directed Evolution of rlac1338

#### Error-Prone PCR to Generate Mutant Laccase Gene and Construction of Mutant Library

Using mutant primers Mut-F and Mut-R, a mutant gene fragment with *Hind* III and *Bam*HI restriction sites was amplified, the size was about 1.3 kbp, and the target strip was recovered by 1% agarose gel electrophoresis band. Randomly picked clones and extracted plasmids for double enzyme digestion, and subjected the digested products to gel electrophoresis analysis. According to statistics, the number of clones in the library can reach 10^8^, which meet the requirements of library screening.

#### Primary Screening of Highly Active Laccase Mutants

After being cultured, the positive transformants were developed with ABTS, and the enzyme activity was judged by the intensity of the color, that is, the darker the color, the higher the enzyme activity (Supplementary Figure 4). The results showed that about 40% of the mutants had no color change or lighter color than the wild-type laccase, about 40% of the activity was basically unchanged, and only about 20% of the mutants had an increase in activity. It can be seen that the mutations affected the laccase enzyme active.

#### Rescreening of Highly Active Laccase Mutants

**FIGURE 1:**
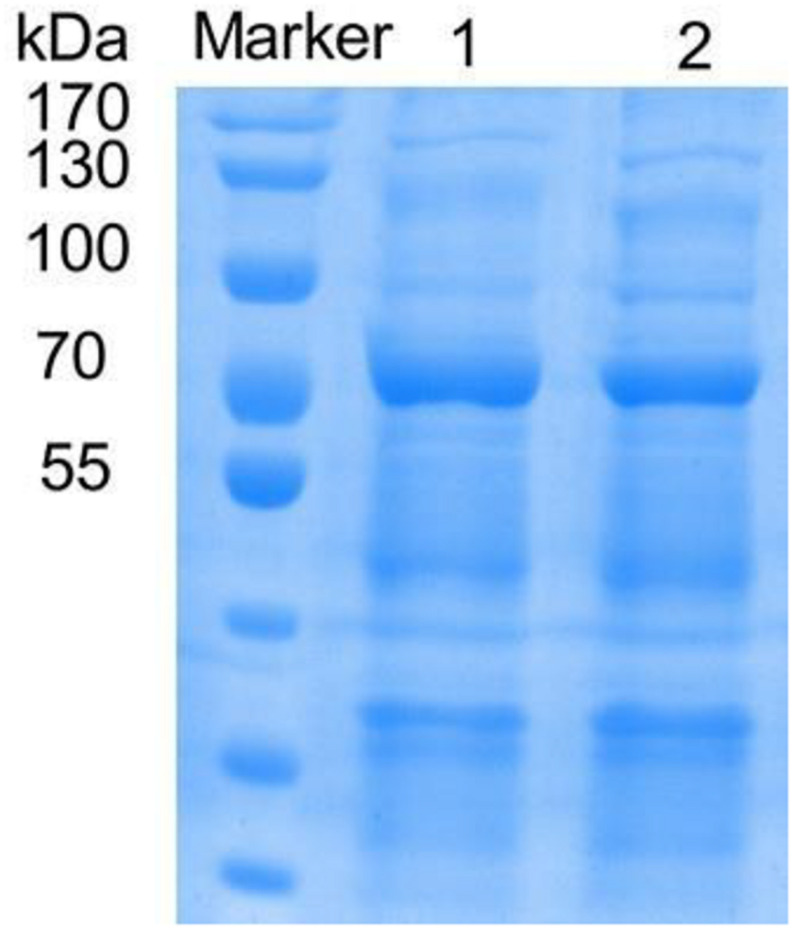
SDS-PAGE of recombinant and mutanted laccase. M, TaKaRa; 1, recombinant laccase *lac*1338; 2, Mutanted laccase.

The mutants with higher activity obtained in the preliminary screening were identified by SDS-PAGE electrophoresis, and the results showed that the size of the laccase protein produced by the mutants was similar to that of the rlac1338 (Figure 1).

#### Mutant Gene Sequence Sequencing

The strains with higher enzyme activity than the wild type were obtained by functional screening (Xia et al., 2014; Lv et al., 2015), which were named lac1-16, lac1-19, lac2-1, and lac2-9 after sequencing (Table 1). Obviously, the enzyme activity of the mutant strain lac2-9 was significantly improved.

**TABLE 1:** Nucleotide substitution and amino acid changes of the mutant enzymes.

**Mutant**	**Mutant base**	**Mutant amino acid**	**Specific enzyme activity (U/mg)**
rlac1338	–	–	22.8 ± 1.5
1-16	T234C/T258C/A943G	S314G	45.6 ± 2.2^*^
1-19	T496C/T975C	M317T	34.25 ± 1.9^*^
2-1	T72A/A104G	V15D/I26V	34.25 ± 1.4^*^
2-9	T243C/T267C/T552A		
	T842C/C926T/A952G/T698A	V281A/P309L/S318G/D232V	79.8 ± 2.7^*^

^*^Indicates that the data is significant, that is *p* < 0.05.

### Properties of Enzymes

**FIGURE 2:**
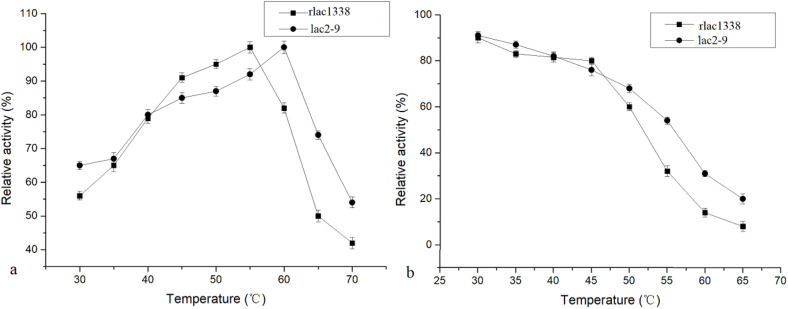
Comparison of optimal temperature and thermal stability between mutant strain lac2-9 and recombinant rlac1338.

**FIGURE 3:**
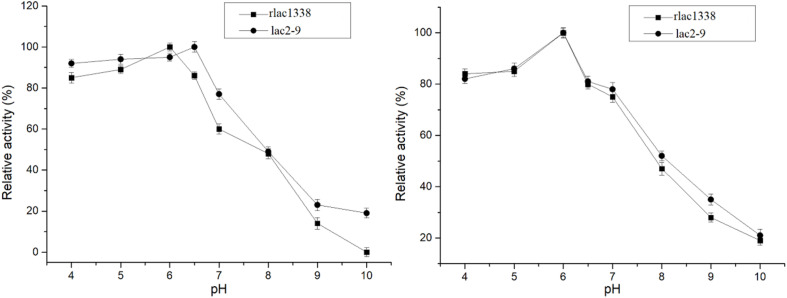
Comparison of optimal pH and pH stability of mutant strain lac2-9 and recombinant rlac1338.

The optimal reaction temperature of the mutant enzyme was 5°C higher than that of rlac1338 (60 vs. 55°C) (Figure 2). The thermal stability was also improved, and the relative enzyme activity was still over 50% after being kept at 55°C for 4 h (Figure 2). The results of pH and pH stability were shown in Figure 3. With ABTS as the reaction substrate, the mutant strain had a pH increase of 0.5 unit compared with the recombinant laccase, but its stability was not significantly different.

### Kinetic Properties of Recombinant Laccase rlac1338 and Mutant lac2-9

The Michaelis constant K_m_ and specific enzyme activity of rlac1338 and mutant lac2-9 for non-phenolic or phenolic substrates were measured at the optimal reaction pH and temperature (Table 2). With ABTS as the substrate, the k_cat_/K_m_ of rlac1338 and mutant strain lac2-9 are the largest than other substrates, 0.1638 and 0.618 s^–1^M^–1^, respectively, indicating that ABTS is the most suitable substrate for the recombinant enzyme and mutant enzyme. In addition, the K_m_ of the mutant strain lac2-9 (76 μM) was significantly lower, but the k_cat_/K_m_ (0.618 s^–1^M^–1^) was significantly higher, and the specific enzyme activity (79.8 U/mg) increased by 3.5 times compared with the recombinant laccase (22.8 U/mg), the specific enzyme activity to other substrates also improved to different degrees compared with the recombinant laccase.

**TABLE 2:** Comparison of kinetic parameters and enzyme activity between mutant enzyme lac2-9 and rlac1338.

**Enzyme**	**Substrate**	**K_m_(μM)**	**k_cat_ (s^–1^)**	**k_cat_/K_m_ (s^–1^μM^–1^)**	**Specific enzyme activity (U/mg)**
rlac1338	2,6-DMP	6542.1	9.430.8	1.44 × 10^–2^	1.120.02
	ABTS	2103.3	34.391.1	0.16	22.801.5
	Guaiacol	4,9001.8	1.340.04	2.7 × 10^–4^	0.640.04
	Catecho	5072.5	13.991.0	2.76 × 10^–2^	10.601.6
	1-naphthol	5,6002.4	3.560.4	6.4 × 10^–4^	0.130.01
lac2-9	2,6-DMP	5603.7	11.231.2	0.02	2.670.05
	ABTS	761.8	46.942.5	0.62	79.803.6
	Guaiacol	4,3001.6	1.570.3	3.7 × 10^–4^	1.370.05
	Catecho	5702.9	30.761.6	5.4 × 10^–2^	18.901.3
	1-naphthol	6,0403.1	3.890.7	6.4 × 10^–4^	8.9 × 10^–2^

**The reaction system of 3 mL citric acid-sodium citrate buffer solution includes 5 mmol/L substrate, 6 mmol/L Cu^2+^, 55°C, 3 min.**

### Comparison of Dye Degradation by lac2-9 and rlac1338

**FIGURE 4:**
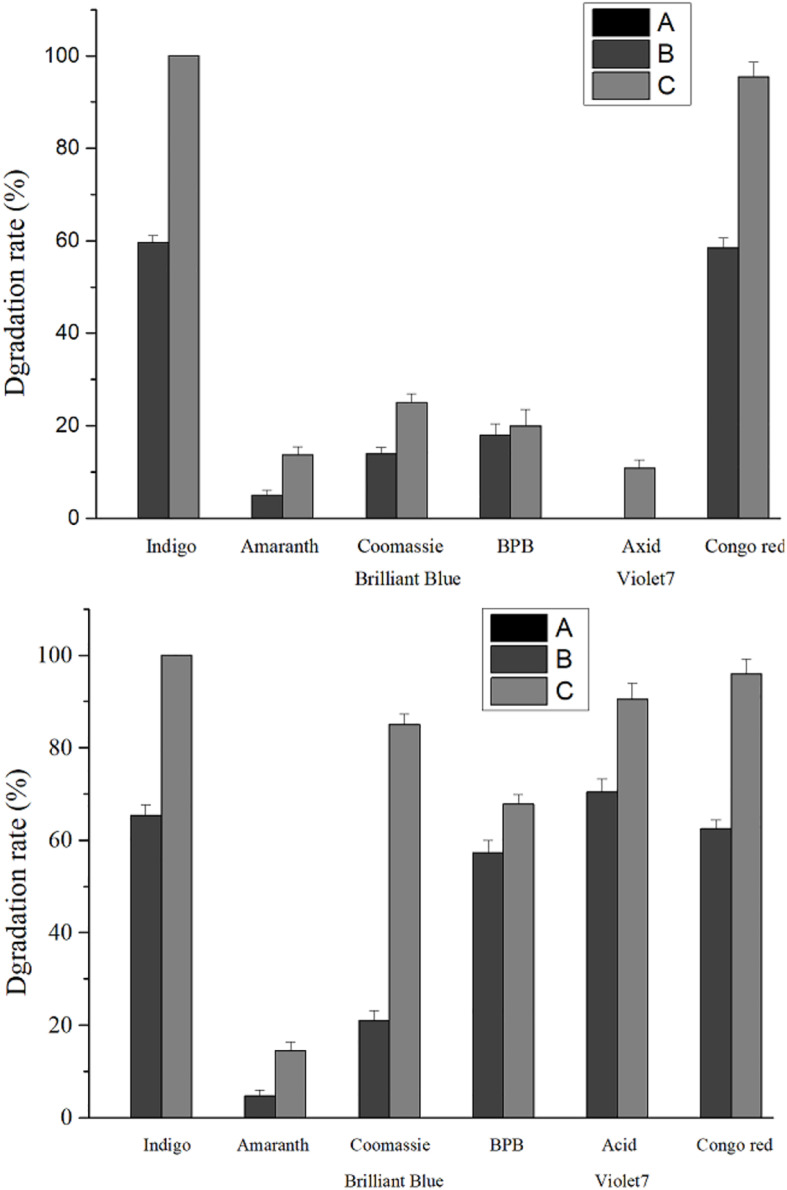
Comparison of dye degradation rate between mutant enzyme lac2-9 and rlac1338 in the presence of small molecule mediator ABTS and Ca^2+^. A, no ABTS and Ca^2+^; B, 100 mM Ca^2+^; C, 100 mM Ca^2+^ and 20 μM ABTS. Measurement conditions: 37°C, decolorization for 24 h.

Compared with the recombinant laccase and rlac1338, the degradation rates with the simultaneous addition of Ca^2+^ and ABTS of mutant strain lac2-9 over acid violet 7, bromophenol blue, coomassie brilliant blue and amaranth increased from 10.9, 20, 25, and 13.7 to 90.5, 67.8, 85, and 14.5%, respectively (Figure 4). Hence, it is feasible to obtain functionally enhanced mutant enzymes through error-prone PCR.

### Homologous Three-Dimensional Structure Model of Mutant Strain

**FIGURE 5:**
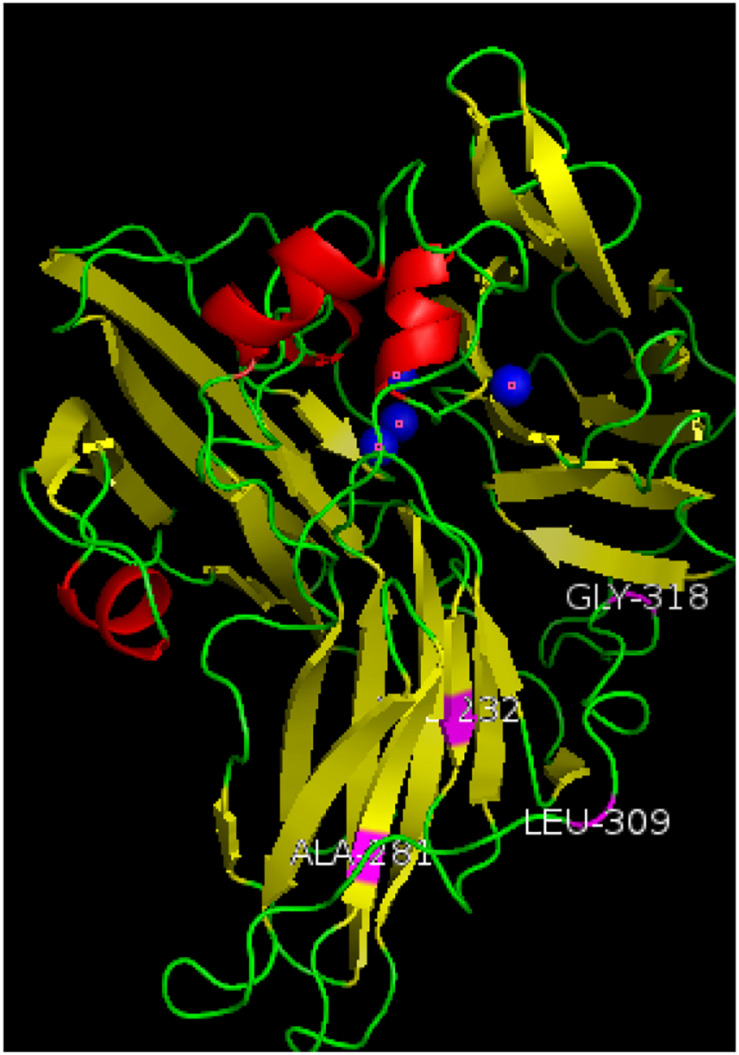
Three-dimensional structure simulation diagram of mutant enzyme lac2-9. α-Helixes (red), β-sheets (yellow), loops (green) and copper atoms (blue) are depicted. The mutation sites of the mutant enzyme (shown in purple in the Figure 5) V281A, P309L, S318G, and D232V are far away from the active center of laccase, and are located on the surface or ring region of the laccase protein. Among them, 309 and 318 are on the loop of the spherical structure, and 281 and 232 are on the α-spiral.

The amino acid sequences of the mutant enzyme and recombinant laccase were submitted to the Phyre2 protein online analysis server (see text footnote 1). Using homology modeling, its three-dimensional structure is similar to Crystal structure of Lac15 from a marine microbial metagenome (4f7k.1.A). Its three-dimensional structure was simulated, and copper in the structure atoms and mutant amino acids was marked using Pymol. Results showed that the enzyme existed in the form of a single subunit protein (Figure 5). The entire monomer molecule was composed of three cupredoxin-like domains (Domains 1, 2, 3), which were divided into 3 regions accordingly. Each domain has a β-barrel shape (the β-ropes are arranged into β-hinges to form the so-called Greek pattern). The amino acid sequences of the mutant enzyme and the recombinant laccase lac1338 were compared. The mutation sites (shown in purple in the figure) V281A, P309L, S318G, and D232V are far from the active center of the laccase, and are located on the surface or loop region of the laccase protein.

## Discussion

The gene encoding codon optimized *lac1338* was synthesized. The biggest advantage of this method is that the protein can be expressed at a high level. The optimum reaction temperature of most fungal laccases discovered so far is between 25 and 50°C. Zhao (2012) screened a white rot fungus with high laccase production, the optimum temperature of the laccase produced by the bacteria is 30°C. However, bacterial laccase generally has a relatively high optimal reaction temperature. Li et al. (2020) selected a strain of laccase from *Bacillus thuringiensis* that produces a high-temperature laccase, and its optimal temperature is 75°C. The optimum temperature of the laccase synthesized in this experiment is 55°C, and the optimum temperature of lac2-9 is 60°C. Although it is lower than some bacterial laccases, it is also higher than most fungal laccases. This may be an advantage for the application of this laccase in industrial production in high temperature environment.

At present, directed evolution methods mainly include error-prone PCR, DNA shuffling, random *in vitro* recombination, and staggered extension (Wang et al., 2017). Directed evolution technology belongs to the category of non-rational design. It does not need to know the three-dimensional structure information and mechanism of protein in advance. Instead, it creates a process similar to natural evolution *in vitro*, causes a large number of mutations in genes, and then selects the required ones through specific screening methods. The nature or function of the target gene. In particular, it has certain advantages in modifying the enzymatic properties of enzymes, such as improving the thermal stability of the enzyme, expanding the range of substrates, making the optimal reaction pH more acidic or alkaline, and improving enzyme activity. Directed evolution is usually divided into three steps: the first step is to generate diverse genes through random mutation or *in vitro* recombination; the second step is to construct a mutation library after the mutated gene is introduced into an appropriate vector; the third step is to select by appropriate screening methods Mutants of desired properties. The whole process can be cycled many times until the enzyme with the desired properties is obtained. The error-prone PCR technology selected in this study is a relatively simple and fast random mutation strategy that only needs to change a single condition to produce greater mutation benefits. After the mutation library is constructed, the choice of an efficient and sensitive screening method is the success of directed evolution to transform the protein molecule. According to laboratory conditions, by simulating the activity screening method of 96-well plates, the apparent enzyme activity of the mutants is preliminarily determined based on the color reaction of the substrate, that is, the faster the color reaction speed and the darker the color, the higher the corresponding mutant laccase enzyme activity; the detection of enzyme activity in the re-screening process avoids misjudgment and improves the accuracy and authenticity of the screening results. Explore the amino acids that affect enzyme activity and stability through the three-dimensional structure, and the results show that in the mutation sites of the mutant enzyme lac2-9, positions 281 and 309 were replaced by non-polar amino acids with polar amino acids, and position 318 was mutated from the neutral amino acid serine to the non-polar amino acid glycine. These positions may be involved in maintaining the spatial conformation required for laccase catalytic oxidation. The mutation at position 232 from the polar positively charged aspartic acid to the non-polar amino acid valine improves the surface hydrophobic effect on the mutant enzyme molecule, making it more suitable for binding to the substrate at high temperatures (Autore et al., 2009), and affects the combination of T2/T3 copper trinuclear active center and hydroxide radical, so that the optimal pH changes (Madzak et al., 2006). Therefore, the mutation at this site improves the optimum temperature and stability of the mutant enzyme.

Studies show that the mutant enzyme has improved dye degradation types and degradation rates compared to the recombinant laccase rlac1338, indicating that it directed evolution is feasible for obtaining a mutant enzyme with more industrial application value. Miele et al. (2010) improved the enzyme activity of *Pleurotus ostreatus* laccase POXA1b by directed evolution, which increased the degradation rates of acid yellow 49, acid red 266 and direct yellow 106. By increasing the enzymatic activity of the laccase CotA of *Bacillus licheniformis*, Koschorreck et al. (2009) enhanced the degradation effects of the laccase on the dyes such as alizarin red S, brilliant blue R, and isatin. But laccase can only degrade phenolic dyes, but cannot directly oxidize non-phenolic dyes. Studies have found that after adding some small molecule compounds called laccase mediators, it can promote the catalytic effect of laccase on non-phenolic substrates, and can significantly improve the catalytic efficiency of phenolic substrates, thereby further expanding laccase the scope of the substrate. These mediators themselves are also laccase substrates, acting as electron transfer intermediates, allowing electrons to be transferred between the enzyme and the substrate. At present, the most commonly used synthetic mediator is 2,2-azide-bis (3-ethylbenzothiazole-6-sulfonic acid) (ABTS). Laccases from various sources can quickly oxidize ABTS to ABTS^+^ intermediate body, and then oxidize the substrate. ABTS can enhance the dye degradation efficiency of laccase, and mediate the oxidation between laccase and non-enzymatic substrate dyes, so that laccase can degrade the dyes of non-laccase substrates (Singh and Kumar, 2010). For example, azo dyes are not the substrates of laccase, and cannot be directly degraded by most laccases (Camarero et al., 2005). Nevertheless, rlac1338 can completely degrade the azo dye rhodamine under the synergistic effect of ABTS and Ca^2+^. Laccase lac1338 has a pI of 5.05, and its optimum pH is 6.0, so the laccase protein has a positive charge. Ca^2+^ is a positive ion, which binds to the dye anion, which reduces the charge repulsion between the laccase protein and the dye ion, and improves the rate at which the enzyme adsorbs the dye molecules (Sheng, 2019). In the next work, we will combine the three-dimensional structure of the laccase protein and its bioinformatics to further explore the reasons why the mutant base changes its properties.

## Data Availability Statement

The original contributions presented in the study are included in the article/Supplementary Material, further inquiries can be directed to the corresponding author/s.

## Author Contributions

SD: conceptualization, original draft preparation, and editing. QY: manuscript reviewing. GY, SL, JY, and XX: supervision. HL and ZD: project administration and supervision. All authors contributed to the article and approved the submitted version.

## Conflict of Interest

SL, JY, and XX were employed by company Guangzhou Base Clean Cosmetics Manufacturer Co., Ltd. The remaining authors declare that the research was conducted in the absence of any commercial or financial relationships that could be construed as a potential conflict of interest.

## Footnotes

1 http://www.sbg.bio.ic.ac.uk/
