# Supplementary material for: Biochemical Characterization of a Novel Bacterial Laccase and Improvement of Its Efficiency by Directed Evolution on Dye Degradation
Source: Front Microbiol. 2021 May 12;12:633004. doi: 10.3389/fmicb.2021.633004 (PMC8149590; doi:10.3389/fmicb.2021.633004)
Supplement: Supplementary file 1 [file Data_Sheet_1.PDF]

```
                1        10        20        30        40        50        60
lac1338         MNRRDFLVTTSAATLFPRIALASAPVELVAQPVNAQILPEGEPATPMLGFNGGTPGPVLR
4F7K_A          ............................................................
WP_106610581.1  ............................................................
WP_035257147.1  ............................................................
WP_058289265.1  ............................................................
WP_054463654.1  ............................................................
MBA84203.1      ............................................................
MBT24881.1      ............................................................
OIQ43611.1      ............................................................
WP_167685249.1  ............................................................

                         70        80        90       100       110       120
lac1338         ARQGEVFDIRFQNQIGEGSAVHWHGLRIDNAMDGVPGMTQDVVEAGGEFEYSFRAPDAGT
4F7K_A          ..........................................VEAGGEFEYSFRAPDAGT
WP_106610581.1  ....................VHWHGLRIDNAMDGVPGMTQDVVNAGGEFEYRFRAPDAGT
WP_035257147.1  ....................VHWHGLRIDNAMDGVPGMTQEVVEAGDGFEYRFRAPDAGT
WP_058289265.1  ....................VHWHGLRIDNAMDGVPGLTQDVVEVDAEFAYRFRAPDAGT
WP_054463654.1  ....................VHWHGLRSDNAMDGVPGLTQDVVEAGSEFAYRFRAPDAGT
MBA84203.1      ....................VHWHGLRIDNTMDGVPGMTQGVVSAGDAFAYRFRAPDAGT
MBT24881.1      ...........................................................T
OIQ43611.1      ....................VHWHGLRINNAMDGVPGMTQDVVEKGGELGYRFRAPDAGT
WP_167685249.1  ....................VHWHGIRSDNAMDGVPGLTQDIVQSGDHFDYRFRAPDAGT

                         130       140       150       160       170       180
lac1338         FWYHSHNRSWEQVAKGLYGPLIVEEPTPPDVDHDLIIMIDDWRITENGVLADGFENMRDQ
4F7K_A          FWYHSHNRSWEQVAKGLYGPLIVEEPTPPDVDHDLIIMIDDWRITENGVLADGFENMRDQ
WP_106610581.1  FWYHSHNRSWEQVAKGLYGPLIIEEPTPPDVDHDLIVMIDDWRMTENGTLADGFENMHDQ
WP_035257147.1  FWYHSHNRSWEQVAKGLYGPLIVEEPTPPDVDHDLIIMIDDWRMTEDGTLADGFENMHDQ
WP_058289265.1  FWYHSHNRSWEQVAKGLYGPLIVEELTPPDVDHDLIVLIDDWRMTEAGTLADGFENMHDQ
WP_054463654.1  FWYHSHNRSWEQVAKGLYGPLIVDEPTPPDVDHDLIVMIDDWRMTEAGTLAEGFENMHDQ
MBA84203.1      FWYHAHNRSWEQVARGLYGPLIVEEQTPPDVDHDLIVMIDDWRMTDSGTLADDFDNRHDQ
MBT24881.1      FWYHAHNRSWEQVARGLYGPLIVEEQTPPDVDHDLIVMIDDWRMTDSGTLADDFDNRHDQ
OIQ43611.1      FWYHSHNRSWEQVAKGLYGALIVEEPTPPDVDHDLVVTIDDWRMTESGALADDFENMHDQ
WP_167685249.1  FWYHSHNRSWEQVAKGLYGPLIVEENKPPDVDHDITVLIDDWRISVEGVLAGDFDNMHDQ

                         190       200       210       220       230       240
lac1338         AHQGRLGNFARALVEPVTPVRRGDRVRLRLINVATDRIFPVELEGVEGKVVALDGMPIVD
4F7K_A          AHQGRLGNFARALVEPVTPVRRGDRVRLRLINVATDRIFPVELEGVEGKVVALDGMPIVD
WP_106610581.1  AHQGRLGNFARAHVEPVRQVRRGDRVRLRLINVATDRIFPVELEGVEGKVVALDGMPLES
WP_035257147.1  AHQGRLGNFARALVEPVTQVRRGDRLRLRLINVATDRIFPVELEGVEGKVVALDGMPLVT
WP_058289265.1  AHQGRLGNYARALVEPSTPVRRGDRVRLRLINVATDRIFPIEIEGVDGKIVANDGMPLVS
WP_054463654.1  AHQGRLGNFARALVEPQVTVRRGDRVRLRLINVATDRIFPVEIEGIDGKIVALDGMPLSD
MBA84203.1      AHQGRLGNFARALIVPSTPARRGDRLRLRLINVATDRIFPVELEGIDGKVVALDGMPLGR
MBT24881.1      AHQGRLGNFARALIVPSTPARRGDRLRLRLINVATDRIFPVELEGIDGKVVALDGMPLGR
OIQ43611.1      AHQGRLGNFARALVDPNTPVRFGDRLRLRLINVATDRIFPVELEGIEGKVVALDGMPLVS
WP_167685249.1  AHAGRMGNFARAIINTKAPVQTRDRLRLRLINVATDRIFPIDLIGIEGKVVALDGMPLAE

                         250       260       270       280       290       300
lac1338         PQEFSGLILAPAQRADIIADVITDAPIGFVFPTRDGPYLLGEIPVKGANTTRQPSEIPAL
4F7K_A          PQEFSGLILAPAQRADIIADVITDAPIGFVFPTRDGPYLLGEIPVKGANTTRQPSEIPAL
WP_106610581.1  LQELSGLILAPAQRADIIADVTSATSASFAFPTRDGLYLLGEIPVEGSNITRQPSEISTL
WP_035257147.1  PQEMSGLILAPAQRADIIADVTSATSASFVFPTRDGPYLLGEIPVEGANTTRQPSEISVL
WP_058289265.1  PQELSSLILAPAQRADIIADITSPDQIAFMFPTQDGPYLLGEIPVEDANTERQPSEISAL
WP_054463654.1  PHELSDFILAPAQRVDIIADVATSDQVAFVFPTRDGPYLLGEVPVAGENTERQPSVVRAL
MBA84203.1      PQELSGLVLAPAQRADIIADVVSADPIAFVFRTQDGPYLLGEIPVEGVNSMRQASEISAL
MBT24881.1      PQELSGLVLAPAQRADIIADVVSADPIAFVFRTQDGPYLLGEIPVEGVNSMRQASEISAL
OIQ43611.1      PQELSDLVLAPAQRADIIVDVVSANSVSIFLPTRDAPYLLGEIQVEGKNTRRQPTDIPTL
WP_167685249.1  PQDISGLVLAPAQRADIIADVTSSDNVQFVFPTRDDPYVLGEIALEGTNNNRAASDILPL

                         310       320       330       340       350       360
lac1338         PPNEVTSPDMGSAVSLTLTMEGGAMSRRMMQGMMGGDIWAFNGQSGLTDTPLHSFERGQT
4F7K_A          PPNEVTSPDMGSAVSLTLTMEGGAMSRRMMQGMMGGDIWAFNGQSGLTDTPLHSFERGQT
WP_106610581.1  TQNEVASPDMDNAISLTLTMEGGAMSRRMMQGMMDWNIWAFNGQSGLTDTPLHRFERGQT
WP_035257147.1  TPNEIASPDMDNAVSLTLTMEGGAMSRRMMQGMMGGDIWAFNGQSGLTDTPLHSFERGKT
WP_058289265.1  LPNEVTQPDLDNAVSLTLTMEGGAMSRRMMQGMMGGDIWAFNGQSGLTDTPFHLFERGQT
WP_054463654.1  PANETAQPDMGNAIPLTLTMQGGAMSRRMMQGMAGGGIWAFNGQSGLTDTPFHLFERGQT
MBA84203.1      PPNEMAQPDMDNAVSLTLSMEGGAMSRRMMQGMMGGNIWAFNGQSGLTDTPFHAFERGQT
MBT24881.1      PPNEMAQPDMDNAVSLTLSMEGGAMSRRMMQGMMGGNIWAFNGQSGLTDTPFHAFERGQT
OIQ43611.1      PPNKAVRPDMGNAVSLTLTMEGGAMSRRMMQGMMGGEIWALNGQSGLKDTPFHSFENGQT
WP_167685249.1  PVNTMAQPDLDNVISLTLTMEGGAMSPRSSS...LTDIWGFNGQSGLNDAPFHSFERGQT
```

```
                         370       380       390       400       410       420
                           .         .         .         .         .         .
lac1338         ARIRLVNDTRFPHGIHLHGHHFFEVGADGNLGALRDTTLVDAGETRDIVCVFDNPGNWLL
4F7K_A          ARIRLVNDTRFPHGIHLHGHHFFEVGADGNLGALRDTTLVDAGETRDIVCVFDNPGNWLL
WP_106610581.1  ARIRLVNDTRFPHGIHLHGHHFFELGLDGSQGAFRDTTLVDAGKSRDIVCVFDNPGRWLL
WP_035257147.1  ARIRLVNDTRFPHGIHLHGHHFFEVGADGNLGALRDTTLVDADETRDIVCVFGNPGRWLL
WP_058289265.1  ARIRLVNDTRFPHGIHLHGHHFFEVRADNNLGAFRDTTLVDAGEARDIVCVFDNPGKWLL
WP_054463654.1  ARIRLVNDTSFAHGIHLHGHHFFEVEADGDLGALRDTTLVDAGETRDIVCVFDNPGRWLL
MBA84203.1      ARIRLVNDTRFPHGIHLHGHHFFEVETDGTLGALRDTTLVDAGESRDIVCVFDNPGKWLL
MBT24881.1      ARIRLVNDTRFPHGIHLHGHHFFEVETDGTLGALRDTTLVDAGESRDIVCVFDNPGKWLL
OIQ43611.1      ARIRLVNDTRFAHGIHLHGHHFFEVGGDDSLGAFRDTTLVEPGETRDIVCVFDNPGKWLL
WP_167685249.1  ARISMVNDTRFPHGIHLHGHHFFEVQADGMLGAFRDTTLVTPGETRDILCIFDNPGKWLL
```

```
                         430
                           .
lac1338         HCHMLGHQAAGMKTWVEVA........
4F7K_A          HCHMLGHQAAGMKTWVEVALEHHHHHH
WP_106610581.1  HCHMLGHQAAGMKTWVEVV........
WP_035257147.1  HCHMLGHQAAGMKTWVEVA........
WP_058289265.1  HCHMLGHQAAGMKTWVEVA........
WP_054463654.1  HCHMLGHQAAGMKTWVEVV........
MBA84203.1      HCHMLGHQAAGMKTWVQVT........
MBT24881.1      HCHMLGHQAAGMKTWVQVT........
OIQ43611.1      HCHMLGHQAAGMKTWVEVV........
WP_167685249.1  HCHMLGHQAAGMKTWLEVS........
```
