# Supplementary material for: Biochemical Characterization of a Novel Bacterial Laccase and Improvement of Its Efficiency by Directed Evolution on Dye Degradation
Source: Front Microbiol. 2021 May 12;12:633004. doi: 10.3389/fmicb.2021.633004 (PMC8149590; doi:10.3389/fmicb.2021.633004)
Supplement: Supplementary file 2 [file Data_Sheet_2.docx]

**Supporting Information**

**Exploring the effect of laccase modified by PCR on dye degradation**


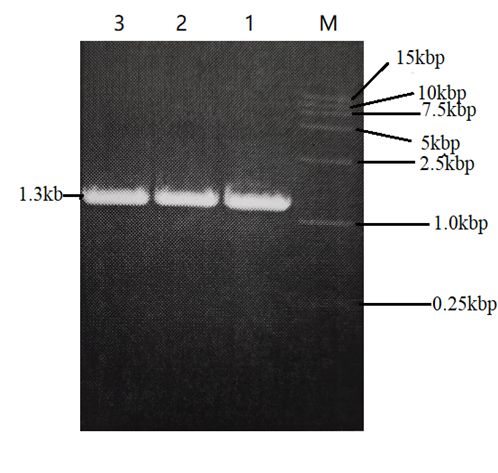


M: DL15 000 Make; 1-3: PCR products of lac1338

Figure S1. Electrophoresis analysis of PCR amplified products of lac1338


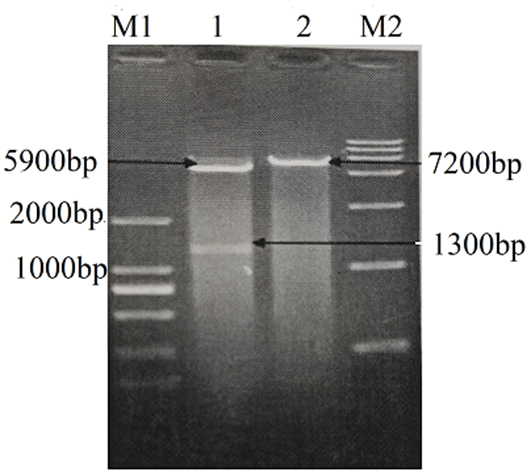


1: Recombinant plasmid digested by *Hind* III and *Bam*H I;

2: Recombinant plasmid digested by *Hind* III

Figure S2. Restriction digestion verification of recombinant plasmid pET-32a-lac1338


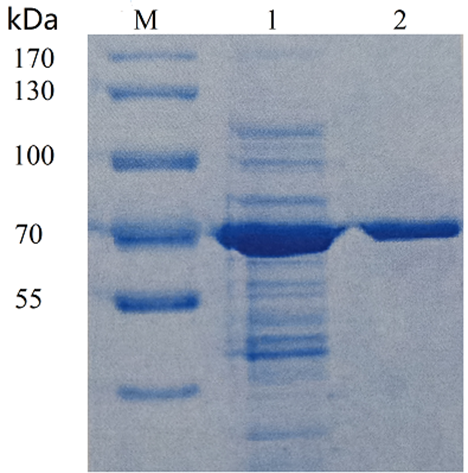


M: Marker; 1: unpurified rlac1338; 2: purified rlac1338

Figure S3. SDS-PAGE electrophoresis of recombinant laccase rlac1338 after purification
